# Short-term mercury exposure disrupts muscular and hepatic lipid metabolism in a migrant songbird

**DOI:** 10.1038/s41598-022-15680-y

**Published:** 2022-07-06

**Authors:** Chad L. Seewagen, Cory R. Elowe, Alexander R. Gerson, Derrick J. E. Groom, Yanju Ma, Mustafa Yildirim, Christopher G. Guglielmo

**Affiliations:** 1 Great Hollow Nature Preserve and Ecological Research Center, New Fairfield, CT USA; 2 Department of Biology, University of Massachusetts - Amherst, Amherst, MA USA; 3 Guangxi Key Laboratory of Rare and Endangered Animal Ecology, Guangxi Normal University, Guilin, Guangxi China; 4 Department of Biology, Advanced Facility for Avian Research, University of Western Ontario, London, ON Canada

**Keywords:** Animal migration, Fat metabolism, Animal physiology

## Abstract

Methylmercury (MeHg) is a global pollutant that can cause metabolic disruptions in animals and thereby potentially compromise the energetic capacity of birds for long-distance migration, but its effects on avian lipid metabolism pathways that support endurance flight and stopover refueling have never been studied. We tested the effects of short-term (14-d), environmentally relevant (0.5 ppm) dietary MeHg exposure on lipid metabolism markers in the pectoralis and livers of yellow-rumped warblers (*Setophaga coronata*) that were found in a previous study to have poorer flight endurance in a wind tunnel than untreated conspecifics. Compared to controls, MeHg-exposed birds displayed lower muscle aerobic and fatty acid oxidation capacity, but similar muscle glycolytic capacity, fatty acid transporter expression, and PPAR expression. Livers of exposed birds indicated elevated energy costs, lower fatty acid uptake capacity, and lower PPAR-*γ* expression. The lower muscle oxidative enzyme capacity of exposed birds likely contributed to their weaker endurance in the prior study, while the metabolic changes observed in the liver have potential to inhibit lipogenesis and stopover refueling. Our findings provide concerning evidence that fatty acid catabolism, synthesis, and storage pathways in birds can be dysregulated by only brief exposure to MeHg, with potentially significant consequences for migratory performance.

## Introduction

Environmental contaminants are a leading driver of habitat degradation and biodiversity decline around the world^1,2^. Among them, mercury is a ubiquitous pollutant due to widespread releases into the environment from coal combustion, gold mining, cement production, and other anthropogenic activities since the industrial revolution^3^. Mercury emissions have recently declined in some world regions, but globally remain 450% greater than pre-industrial levels and a persistent hazard to human and wildlife health^3^.

Inorganic mercury emitted from anthropogenic sources is readily converted to the more bioavailable and toxic form, methylmercury (MeHg), under anoxic conditions associated with wetlands and other aquatic environments, but MeHg can also be prevalent throughout terrestrial systems and their food webs^4,5^. This places wildlife across a broad range of habitat associations and foraging guilds at risk of exposure to bioaccumulated, harmful levels of MeHg^6^. Among birds, sublethal MeHg exposure is capable of disrupting nervous, endocrine, and immune system function, often manifesting in behavioral abnormalities and reproductive impairments^7^. Wide-ranging adverse effects from MeHg have been documented across many avian taxa, including seabirds, wading birds, waterfowl, shorebirds, raptors, and songbirds, in both the laboratory and the wild^6,7^.

Most research on the effects of MeHg on birds has focused on physiology and behavior in relation to reproduction while migration and other life-history events have been comparatively overlooked^8–10^. Migration is often the deadliest phase of a migratory bird’s annual cycle, making it critical to the conservation of migratory species to understand what and how environmental changes affect migration success^11–13^. Breeding ground exposure of Neotropical migratory songbirds to MeHg was recently shown to be associated with a reduced likelihood of return the following year^10^, which represents the first evidence to suggest MeHg might interfere with one or more of the three main requisites for successful long-distance migration–accurate orientation, strong flight endurance, and rapid stopover refueling. Mechanisms by which MeHg has the potential to affect each of these aspects of bird migration have been proposed^9^, but MeHg has not been found to impact orientation^14^ or refueling performance^15,16^ in the few studies that have been conducted so far. However, MeHg reduced the endurance of yellow-rumped warblers (*Setophaga coronata*) flown in a wind tunnel^17^ and the peak metabolic rates of zebra finches (*Taeniopygia guttata*) exercised in a hop-hover wheel^18^, potentially signaling disruptive effects of MeHg on the metabolic processes that power both short- and long-duration flight.

Fat is the primary fuel birds use during migratory flights because it is about 8–10 times more energy-dense than carbohydrates on a wet mass basis and therefore more economical to transport on the wing^19^. The ability of birds to sustain intense exercise with fat rather than carbohydrates is largely achieved through the upregulation of high concentrations of enzymes and transport proteins that facilitate rapid mobilization, delivery, cellular uptake, intracellular transport, and mitochondrial oxidation of extramuscular fatty acids^20–22^. These proteins have been suggested to be vulnerable to interference from MeHg, which could constrain the long-distance flight abilities of migratory birds^9^. The physiological processes that facilitate rapid fattening for migration are similarly thought to have the potential to be inhibited by MeHg exposure^9^. Yet, despite growing evidence from humans and non-avian animal models that MeHg is a metabolic disruptor^23–26^, the effects of MeHg on lipid metabolism pathways that support endurance flight and stopover refueling have never been studied in birds. Here, we examined the effects of short-term, environmentally relevant MeHg exposure on multiple markers of lipid metabolism in the pectoralis muscles and livers of wild-caught, migratory yellow-rumped warblers to test the prediction that MeHg interferes with avian energy metabolism^9^, and investigate potential mechanisms underlying reduced exercise performance in MeHg-exposed birds^17,18^.

## Methods

### Animal care and MeHg exposure

We analyzed tissues that were collected from 24 yellow-rumped warblers used by Ma et al.^17^ to study the effects of MeHg on flight performance in a wind tunnel. The reader is directed to Ma et al.^17^ for detailed methods regarding capture, husbandry, MeHg exposure, and wind tunnel testing. Briefly, the birds were captured during migratory stopovers at Long Point, Ontario, Canada in the autumn of 2014 and transported to indoor aviaries at the University of Western Ontario in London, Canada. There they were maintained on an ad libitum mercury-free, synthetic diet under light cycles that simulated autumn (12L:12D) and then winter (9L:15D) photoperiods until March of 2015, when the photoperiod was lengthened (16L:8D) to stimulate the birds into spring migratory condition. After 14 d of the lengthened photoperiod, 12 randomly selected birds (4 male, 8 female) were switched to an ad libitum synthetic diet containing 0.5 ppm ww MeHg for 14 d while the other 12 birds (3 male, 9 female) continued to feed on the same mercury-free diet to serve as a control group. The exposure concentration of 0.5 ppm ww was intended to represent the middle to upper range of MeHg levels commonly found in the arthropod prey of songbirds in eastern North America^4,27,28^.

Blood samples were collected by brachial venipuncture on days 0 and 14 of the experiment, and stored at − 80º C. Total body mass was measured to 0.01 g on a digital balance, and fat mass and lean mass were measured to 0.001 g with quantitative magnetic resonance analysis, also on days 0 and 14. On day 14, 22 of the 24 birds were flown for up to 2 h in a wind tunnel, after which they were immediately euthanized. Two individuals that could not be flown in the wind tunnel because of missing tail feathers were also euthanized on day 14 for inclusion in the present study. Pectoralis muscles and livers were then collected from all 24 birds immediately following euthanasia and stored at − 80º C. Exposed and control birds did not differ in body size (wing length) upon capture, or body mass or body composition at the start or end of the 14-d experiment^17^.

Bird collection was authorized by the Canadian Wildlife Service (scientific collecting permit SA-0208), and experimental treatments were approved by the University of Western Ontario’s Animal Care Committee (protocol 2010–216) and performed in accordance with all applicable guidelines and regulations.

### Laboratory analyses

#### Tissue mercury concentrations

We measured blood total mercury (THg; ww) as a proxy for MeHg using a direct mercury analyzer, as described in Ma et al.^17^. The instrument was calibrated with a certified reference material (Caprine Blood SRM 955c) and quality control was assessed with a method blank, certified concentration standard (CCS), and sample duplicates. Mean percent recoveries were 104.33 ± 3.37% (SRM 955c; n = 8), 92.15 ± 0.98% (DORM-2; N = 3), and 97.85 ± 0.81% (CCS; N = 36). CVs of duplicates averaged 6.84% ± 1.40%. We estimated pectoralis and liver THg concentrations based on blood THg concentrations, using regression equations from Ma et al.^17^.

#### Metabolic markers

We measured citrate synthase (CS) and carnitine palmitoyltransferase (CPT) activity to assess aerobic capacity and fatty acid oxidation capacity, respectively^29–31^. We measured the gene expression of fatty acid transport proteins to assess muscular and hepatic fatty acid uptake capacity. They included fatty acid translocase (FAT/CD36) and plasma membrane fatty acid binding protein (FABPpm), to evaluate membrane fatty acid uptake capacity, and heart-type fatty acid binding protein (H-FABP), to evaluate intracellular fatty acid transport capacity (pectoralis only)^20,21,30^. We then measured expression of peroxisome proliferator-activated receptors (PPAR-α, PPAR-ß, PPAR-*γ*), which are nuclear receptors that regulate lipid metabolism in birds^32^. PPAR-α and PPAR-β are highly expressed in heart and skeletal muscle where they mainly target genes for proteins involved in lipid oxidation, including CPT and FAT/CD36^33,34^. PPAR-γ, in contrast, primarily controls expression of FABPpm, FAT/CD36, and lipogenic enzymes in the liver and adipocytes to regulate lipid synthesis and storage^33,34^. Lastly, we measured lactate dehydrogenase (LDH) activity to assess effects of MeHg on glycolytic capacity given the broad ability of MeHg to inactivate enzymes^35^ and the effect this could have on migrating birds early in flight, before they switch over from catabolizing primarily carbohydrates to primarily fatty acids^36^.

#### Enzyme assays

We homogenized 50–100 mg of pectoralis and liver using beadmill homogenization (speed 8, time 3 for liver and time 4 for pectoralis, 4 °C) in 9 volumes of 20 mM Na_2_PO_4_, 0.5 mM EDTA, 0.2% BSA, 0.1% Triton x-100, and 50% Glycerol, pH = 7.4. We then assayed CS, CPT, and LDH in duplicate or triplicate in a microplate reader at 39 °C to represent a typical avian body temperature. We measured LDH at 340 nm using 0.4 mM pyruvate, 0.66 mM NADH, 5 mM DTT, 50 mM Tris, pH = 7.4. For CS and CPT, we measured absorbance at 412 nm to detect the appearance of CoA-TNB as in Price et al.^37^.

#### qPCR of transport proteins and PPARs

We homogenized ~ 50 mg of pectoralis and liver in 1 mL of TriZol (Invitrogen, Carlsbad, CA) and separated RNA into an aqueous phase using a chloroform ethanol procedure. We purified total RNA using a PureLink RNA kit (Invitrogen, Carlsbad, CA) and quantified RNA using a plate adapter. We treated 14 µg of RNA using TURBO I DNase (Invitrogen, Carlsbad, CA) and used Nanodrop 2000 (Thermo Fisher Scientific, Waltham, MA) to quantify and check quality before reverse transcription of 1 μg of DNase-treated RNA using NEB Luna RT (New England Biolabs, Ipswich, MA).

We selected Glyceraldehyde 3-phosphate dehydrogenase (*GAPDH*) as a housekeeping gene for qPCR using a published primer sequence for yellow-rumped warblers^38^ and used published primer sequences for FAT/CD36, FABPpm, H-FABP, and PPARs^39–41^. Each cDNA sample was diluted 1:5 with nuclease-free water before analysis. We confirmed primer specificity on a 2% agarose gel and used a serial dilution series of pooled cDNA to obtain amplification efficiency (between 97.1% and 99.9%). Reactions were run in duplicate in a StepOnePlus qPCR machine (Applied Biosystems, Foster City, CA; 60 s at 95 °C, followed by 40 cycles at 95 °C for 15 s and 60 °C for 30 s) in 10 µL volumes with 0.2 µM primers and 1 µL cDNA in Luna Universal qPCR master mix (New England Biolabs, Ipswich, MA). We used comparative cycle threshold (C_T_) analysis following Schmittgen and Livak^42^, subtracting the average C_T_ for the corresponding housekeeping gene reading (*GAPDH*) from the average C_T_ from duplicate target gene wells to obtain the ΔC_T_ for each individual and gene, then subtracting the control group mean ΔC_T_ from the MeHg-exposed group mean to obtain ΔΔC_T_. Fold change was calculated for the mean and upper and lower limits of the SEM using 2^−(ΔΔCT)^.

### Statistical analyses

We compared blood THg concentrations between treatment groups with a two-tailed *t*-test. We tested the effects of treatment on each enzyme, transport protein, and PPAR isoform using general linear models. We included lean mass, fat mass, the lean mass by treatment interaction, and fat mass by treatment interaction in full models which we then compared to nested models with likelihood ratio tests to determine whether any of these terms could be dropped. We retained a term for inclusion in a final model along with treatment when a likelihood ratio test of the full model against the nested model from which it was removed had a P value of < 0.1. We used lean mass also as a proxy for body size (wing length) and sex, as all three variables were significantly related to each other.

We performed all statistical analyses in R (v 4.0.4, R Foundation for Statistical Computing, Vienna, Austria) and accepted significance when *P* < 0.05. We log-transformed non-normal variables prior to analyses and removed outliers that were likely measurement errors, such as unrealistically high or low values, an error in the housekeeping gene, or a consistent error for a set of tissue samples run together. We used -ΔC_T_ values for all statistical analyses of transport protein and PPAR expression data.

## Results

### Tissue THg concentrations

Dietary MeHg exposure for 14 d resulted in a significant difference in blood THg between treatment groups (t_22_ = − 25.58, *P* < 0.0001), with exposed birds averaging 10.1 ppm (± 1.4 SE) and control birds remaining at 0.0 ppm (± 0.0 SE). Estimated muscle and liver THg concentrations averaged 21.9 ppm (± 0.9 SE) and 38.2 ppm (± 1.5 SE), respectively, in the exposed group and 0.0 ppm (± 0.0 SE) in both tissues in the control group.

### Muscle enzymes

**Figure 1:**
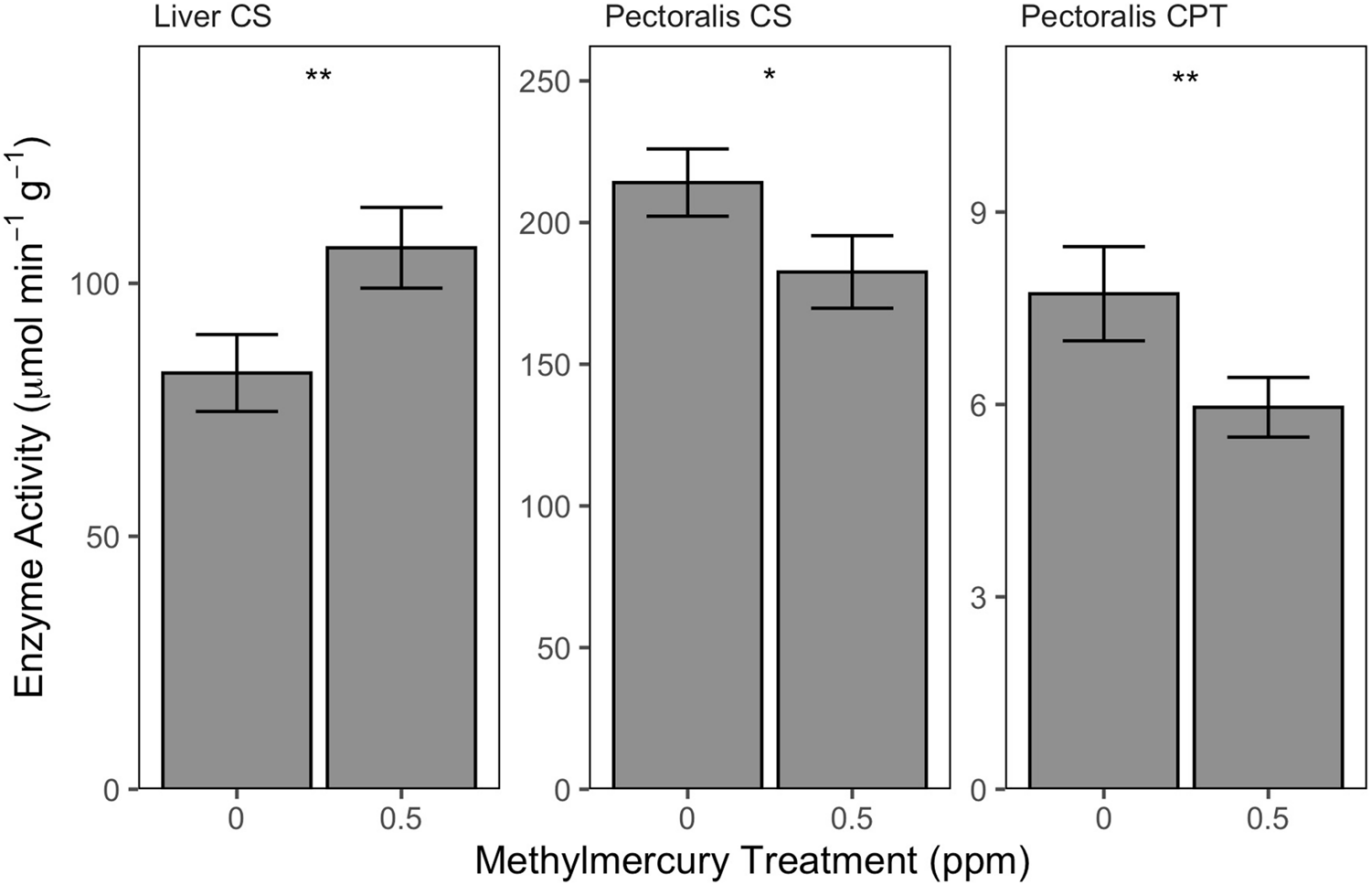
Liver or pectoralis CS and CPT activity in yellow-rumped warblers maintained on a diet with 0.0 ppm or 0.5 ppm methylmercury for 14 d. *Indicates *P* < 0.1; **Indicates *P* < 0.05.

Muscle CPT activity had a negative relationship with lean mass (t_3,20_ = − 2.89, *P* = 0.009); otherwise, no terms were retained with treatment in final muscle enzyme models. Exposed birds had significantly lower muscle CPT activity and trended towards lower muscle CS activity than controls (Table 1, Fig. 1). Muscle CPT and CS activity respectively averaged 23% and 15% lower among exposed birds than controls. There was no difference in muscle LDH activity between treatments (Table 1).

**Table 1:** Muscle and liver enzyme activity, fatty acid transport protein mRNA expression, and PPAR mRNA expression in yellow-rumped warblers maintained on a diet with 0.0 ppm (control) or 0.5 ppm (exposed) methylmercury for 14 d. Enzyme activity (μmol min^−1^ g^−1^) and expression (− ΔC_T_) values are means ± SE, and test statistics are from final general linear models.

	Control	Exposed	t	df	*P*
**Muscle**					
CS	214.1 ± 11.9	182.5 ± 12.8	− 1.807	1, 23	0.085
CPT	7.7 ± 0.7	6.0 ± 0.5	− 2.28	3, 20	0.034
LDH	691.1 ± 76.7	586.9 ± 57.8	− 1.17	1, 23	0.26
FAT/CD36	− 3.4 ± 0.3	− 3.5 ± 0.3	0.34	4, 15	0.74
FABPpm	− 2.2 ± 0.3	− 2.1 ± 0.3	− 0.44	3, 21	0.67
H-FABP	− 1.0 ± 0.3	− 0.8 ± 0.3	0.77	4, 20	0.45
PPAR-α	− 7.5 ± 0.8	− 8.0 ± 0.8	0.24	3, 21	0.82
PPAR-ß	− 5.5 ± 0.6	− 4.3 ± 0.5	− 1.51	2, 22	0.15
PPAR-γ	− 10.7 ± 0.8	− 11.1 ± 0.7	0.42	3, 21	0.68
**Liver**					
CS	82.3 ± 7.6	107.0 ± 8.0	2.25	2, 22	0.035
CPT	10.8 ± 0.6	11.0 ± 0.7	0.17	2, 22	0.87
LDH	1214.1 ± 176.4	1096.2 ± 92.4	− 0.23	2, 22	0.82
FAT/CD36	− 4.1 ± 0.2	− 4.6 ± 0.2	2.16	3, 18	0.045
FABPpm	− 0.2 ± 0.2	− 0.5 ± 0.2	2.93	4, 18	0.009
PPAR-α	− 4.6 ± 0.5	− 5.0 ± 0.7	0.39	3, 19	0.71
PPAR-ß	− 6.3 ± 0.7	− 5.1 ± 0.5	− 1.39	2, 20	0.18
PPAR-γ	− 9.5 ± 0.3	− 10.1 ± 0.3	2.33	4, 17	0.033

### Muscle fatty acid transporters

Muscle FAT/CD36 and H-FABP expression did not differ between treatments (Table 1), with lean mass and fat mass retained in their final models. Fat mass had a positive relationship (t_4,15_ = − 2.30, *P* = 0.036) and lean mass had a marginal negative relationship (t_4,15_ = 2.00, *P* = 0.064) with muscle FAT/CD36 expression in the final model. Fat mass and lean mass also had respective positive and negative relationships with muscle H-FABP expression (fat: t_4,20_ = − 3.67, *P* = 0.002; lean: t_4,20_ = 2.46, *P* = 0.023). There was no treatment difference in muscle FABPpm expression (Table 1). Lean mass was retained in the final model and had a non-significant relationship with FABPpm (t_3,21_ = 1.58, *P* = 0.130).

### Muscle PPARs

Muscle PPAR-α expression did not differ between treatments (Table 1) and had a negative relationship with lean mass in the final model (t_3,21_ = 2.91, *P* = 0.009). There was no difference in muscle PPAR-ß expression between treatments after dropping all other terms (Table 1). Muscle PPAR-*γ* expression did not differ between treatments (Table 1) and had a positive relationship with fat mass in the final model (t_3,21_ = − 2.34, *P* = 0.030).

### Liver enzymes

No terms were retained with treatment in the final models for liver enzymes. Liver CS activity was significantly higher among exposed birds than control birds, by an average of 30% (Fig. 1, Table 1). There were no treatment differences in liver CPT or LDH activity (Table 1).

### Liver fatty acid transporters

**Figure 2:**
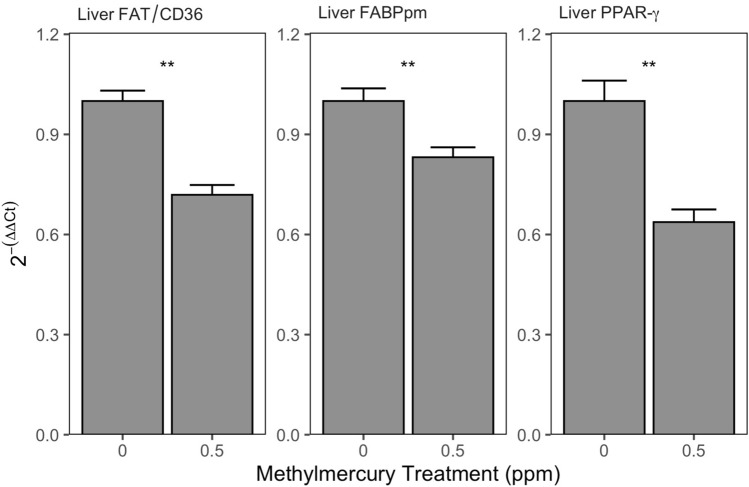
Liver FAT/CD36, FABPpm, and PPAR-*γ* mRNA expression in yellow-rumped warblers maintained on a diet with 0.0 ppm or 0.5 ppm methylmercury for 14 d. **Indicates *P* < 0.05.

Liver FAT/CD36 expression differed between treatments, averaging 12% lower in exposed birds than controls (Table 1, Fig. 2). Lean mass was retained and had a marginal negative relationship with liver FAT/CD36 expression in the final model (t_3,18_ = 1.82, *P* = 0.085). Liver FABPpm expression was also lower among exposed birds (Table 1, Fig. 2), with fat mass and the fat mass by treatment interaction retained in the final model. The interaction between treatment and fat mass was driven by a significant negative relationship between liver FABPpm expression and fat mass among control birds (t_1,9_ = 4.36, *P* = 0.002), while liver FABPpm expression in exposed birds had a non-significant relationship with fat mass (t_1,9_ = − 0.78, *P* = 0.454). Overall, liver FABPpm expression in the exposed birds averaged 2.4-fold lower than in controls (Table 1).

### Liver PPARs

Liver PPAR-α expression did not differ between treatments (Table 1). Lean mass was retained and had a marginal positive relationship with PPAR-α expression in the final model (t_3,19_ = − 1.76, *P* = 0.094). Liver PPAR-ß expression also did not differ between treatments, with all other terms dropped (Table 1). PPAR-*γ* expression in the liver was significantly lower among exposed birds (Table 1, Fig. 2) by an average of 7%, with fat mass and the fat mass by treatment interaction retained in the final model. The interaction between treatment and fat mass was driven by a significant negative relationship between liver PPAR-*γ* expression and fat mass among control birds (t_1,9_ = 2.42, *P* = 0.039), while liver PPAR-*γ* expression in exposed birds had a non-significant relationship with fat mass (t_1,8_ = − 0.66, *P* = 0.530).

## Discussion

We found short-term, environmentally relevant exposure of migrant songbirds to MeHg to have tissue-specific effects on catabolic enzyme activity, fatty acid transport protein expression, and PPAR-*γ* expression, with potentially significant consequences for migratory performance. MeHg-exposed birds indicated lower muscle aerobic and fatty acid oxidation capacity, which may have contributed to their weaker flight performance than controls in a previous study^17^. Livers of exposed birds suggested elevated energy costs, lower fatty acid uptake capacity, and diminished PPAR-*γ* expression, all of which could hinder stopover refueling. Taken together, our results demonstrate for the first time that fatty acid catabolism, synthesis, and storage pathways in migratory birds can be disrupted by MeHg exposure.

### Muscle enzymes

Mercury species have a broad ability to inactivate enzymes by binding to their thiols^35^ and have been observed to inhibit CS and CPT activity or gene transcription in mammals^43^, amphibians^44^, fish^45^, and bacteria^46^. MeHg-exposed birds in our study showed respective decreases of 15% and 23% in muscle activity of CS and CPT-two muscle enzymes that are often substantially upregulated by birds during migration to support endurance flight^39,47–49^. However, there is considerable inter- and intraspecific variation in the degree to which birds adjust muscle catabolic enzyme activity for migration. Dick^49^ found our study species, the yellow-rumped warbler, to respectively increase muscle CS and CPT activity 127% and 270% from winter to spring migration. Other songbirds have been observed to increase muscle catabolic enzymes for migration less dramatically or not at all^29,37,40,50,51^. White-throated sparrows (*Zonotrichia albicollis*), for example, elevated muscle CS and CPT activity 90% each in a field study^39^ and 23% and 57%, respectively, in a photostimulation study^52^. Western sandpipers (*Calidris mauri*) increased muscle CS and CPT activity for migration by only 6% and 13%, respectively^30^. In such cases the proportional decreases we observed in the MeHg treatment would largely or entirely negate the adjustments birds normally make to their muscle enzymatic capacity in support of migration, but it is unknown whether reductions in muscle CS, CPT, or other catabolic enzymes to this extent could have meaningful impacts to flight endurance. The birds in our study indeed had significantly shorter flight durations than control birds in wind tunnel tests^17^, pointing towards the observed reduction in muscle catabolic enzyme activity as a probable cause or contributor. A different wind tunnel study of yellow-rumped warblers failed to find a relationship between CS or CPT activity and flight duration^41^, however, despite substantial increases in CS and CPT activity upon transition to migratory condition^49^. Other metabolic mechanisms acting alone or in combination with reduced muscle CS and CPT activity may therefore have contributed to the weakened flight endurance of our MeHg-treated birds. Inhibition of glycolysis early in flight did not appear to be one such mechanism, as we found no effect of MeHg exposure on muscle LDH.

### Muscle fatty acid transporters and PPARs

The movement of extramuscular fatty acids across muscle cell membranes and through the cytosol is thought to be more rate-limiting than oxidative enzyme capacity in the use of lipids to fuel migratory flight^20–22^. Interference with muscle FAT/CD36, FABPpm, or H-FABP from MeHg would therefore be expected to have greater impacts to flight endurance than oxidative enzyme inhibition^9^. However, we found no effect of MeHg on the yellow-rumped warblers’ expression of flight muscle FAT/CD36, FABPpm, H-FABP, or their PPAR regulators to explain shorter flight durations than control birds in the wind tunnel tests conducted by Ma et al.^17^. We are unaware of any prior studies of the effects of mercury on fatty acid transporters in birds, but fatty acid transporters in other taxa have shown mixed responses. For example, exposure of fish to MeHg increased expression of cytosolic FABP in preadipocytes^53^ and muscles^54^, while it decreased FABPpm expression in preadipocytes^53^, hepatocytes^55^, and kidney cells^45^. FAT/CD36 expression was reduced in adipocytes of mice exposed to mercury chloride^23^. Changes in expression could be due to interactions of mercury with the regulators of fatty acid transporter transcription, such as PPARs, or represent a compensatory response to disruptions of fatty acid transporter function^24,45^. The effects of mercury on fatty acid transporter function appear to be unknown. However, fatty acid transport proteins, including FAT/CD36 and H-FABP, have cysteine residues and associated thiols^56–60^, which make them highly prone to interference from mercury^61,62^. Although we did not observe an effect of MeHg on muscle fatty acid transporter expression, we cannot eliminate the possibility that exposed birds had impaired fatty acid transporter function, and that this hindered their flight performance in the prior study.

### Liver metabolism

While muscles are the center of lipid utilization during flight, the liver plays an essential role in fatty acid synthesis and storage during pre-migratory fattening and stopover refueling^22^. The liver may also provide an important alternative source of fatty acids in the form of very low-density lipoprotein (VLDL) synthesized de novo and released into circulation for storage or fuel^20,22^. MeHg-exposed birds in our study displayed greater metabolic alterations to their liver than flight muscle, possibly because MeHg accumulation in the avian liver is typically double or more than in the pectoralis^17,63,64^. Liver PPAR-*γ* expression in exposed birds averaged 7% lower than in controls. This may have had downstream effects on liver FAT/CD36 and FABPpm expression, which respectively averaged 12% and 2.4-fold lower in the MeHg treatment. We are unaware of other studies of the effects of mercury on PPAR*-γ* (or other PPARs) in birds, but effects in other taxa have been inconsistent. For example, mercury increased PPAR*-γ* expression in nematodes^26^, rat adipocytes^65^, and fish brain cells^66^, while it decreased it in mouse fibroblasts and adipocytes^23,67^, and had no effect in fish preadipocytes^53^. Interestingly, Corder et al.^68^ found liver expression of PPAR*-γ* and target genes for FAT/CD36, FABPpm, and cytosolic FABP in migratory gray catbirds (*Dumetella carolinensis*) to remain similar across the annual cycle, indicating increased expression of PPAR*-γ* and fatty acid transporters may not be needed for the liver to meet the metabolic demands of migration. Similarly, flight training of European starlings (*Sturnus vulgaris*) was found to stimulate expression of multiple PPAR coactivators and genes involved in lipid metabolism in flight muscle but not the liver, also suggesting baseline expression of PPAR*-γ* and its targets outside of the migratory seasons may be sufficient to satisfy demands on the liver during migration^69^. Maintenance rather than upregulation of liver FAT/CD36 and FABPpm expression for migration could also be an adaptive mechanism to limit fatty acid uptake by the liver to spare fatty acids for use by the muscles. Nonetheless, it is conceivable that a 7% reduction in PPAR*-γ* expression, 12% reduction in FAT/CD36 expression, and 2.4-fold reduction in FABPpm expression could too greatly limit the rate at which the liver uptakes fatty acids to synthesize VLDL for migratory fattening (or in-flight fuel). Moreover, PPAR*-γ* regulates key lipogenic enzymes in the liver^70,71^, which are upregulated during migration to facilitate rapid fat storage^48,72^. These enzymes are vulnerable to direct inactivation by mercury^35,73^ in addition to the upstream effects on their PPAR-*γ* regulator. Compound effects of reduced lipogenic enzyme expression and activity from MeHg exposure might slow the pre-migratory fattening and stopover refueling abilities of birds^9^. Detoxification of MeHg with energy that could otherwise be put into storage is yet another mechanism by which MeHg has potential to slow migratory fattening by birds^18^. The higher liver CS activity we observed among exposed birds despite expected countereffects from MeHg^43,46^ suggests a strong response to the high energy costs of depurating MeHg^74^ and a greater reliance on glycogen than fatty acids to fuel those costs given the concomitant decrease in fatty acid uptake capacity.

## Conclusion

The multiple metabolic alterations we observed from only short-term, environmentally relevant MeHg exposure raise concern that global mercury pollution is further challenging what is already often the most perilous event in the annual cycle of migratory birds. In many ecosystems and geographic regions, wild birds are exposed to MeHg at higher levels and/or for much longer periods than those used in our experiment, creating potential for greater metabolic impediments to migratory performance. Also, our study was limited to only some of the many proteins that play key roles in the fatty acid oxidation, synthesis, and storage pathways underlying migration. Additional impacts to flight endurance and refueling ability could result from effects of MeHg on the expression and function of other critical proteins like hormone-sensitive lipase, lipoprotein lipase, long chain acyl-CoA synthetase, albumin, malic enzyme, fatty acid synthase, and hemoglobin^9,23,63,73,75^. There is also the potential for mercury’s neurotoxicity to affect flight biomechanics, as observed in our birds^17^, which could further weaken endurance through reduced flight efficiency^9,17,76^. Much remains to be learned about the effects of MeHg on the physiological systems and processes that give migratory birds the remarkable ability to biannually fly thousands of kilometers between breeding and wintering grounds. Given the significant carry-over effects of migratory performance to population demography and size^11,77,78^, we consider it a priority in migratory bird conservation to better understand the impacts mercury pollution is having on the energetics and survivorship of long-distance migration.

## Data Availability

The data used in this study are publicly available in the Dryad repository (10.5061/dryad.47d7wm3g3).
